# Impact of decitabine on immunohistochemistry expression of the putative tumor suppressor genes FHIT, WWOX, FUS1 and PTEN in clinical tumor samples

**DOI:** 10.1186/1868-7083-6-13

**Published:** 2014-07-03

**Authors:** David J Stewart, Maria I Nunez, Jaroslav Jelinek, David Hong, Sanjay Gupta, Marcelo Aldaz, Jean-Pierre Issa, Razelle Kurzrock, Ignacio I Wistuba

**Affiliations:** 1 University of Ottawa, 501 Smyth Rd, Ottawa, ON K1H 8 L6, Canada; 2 The University of Texas MD Anderson Cancer Center, 1515 Holcombe Blvd, Houston, TX 77030, USA; 3 Fels Institute for Cancer Research and Molecular Biology, 3307 North Broad Street, Philadelphia, PA 19140, USA; 4 UT MD Anderson Cancer Center, 1808 Park Road 1C, Smithville, TX 78957, USA; 5 University of California San Diego, 3855 Health Sciences Dr, La Jolla, CA 92093, USA

**Keywords:** Decitabine, FHIT, FUS1, WWOX, PTEN, Tumor suppressor genes, LINE-1 methylation

## Abstract

**Background:**

Since tumor suppressor gene function may be lost through hypermethylation, we assessed whether the demethylating agent decitabine could increase tumor suppressor gene expression clinically. For fragile histidine triad (*FHIT*), WW domain-containing oxidoreductase (*WWOX*), fused in sarcoma-1 (*FUS1*) and phosphatase and tensin homolog (*PTEN*), immunohistochemistry scores from pre- and post-decitabine tumor biopsies (25 patients) were correlated with methylation of the long interspersed nuclear element-1 (LINE-1) repetitive DNA element (as a surrogate for global DNA methylation) and with tumor regression.

**Results:**

With negative staining pre-decitabine (score = 0), the number of patients converting to positive staining post-decitabine was 1 of 1 for FHIT, 3 of 6 for WWOX, 2 of 3 for FUS1 and 1 of 10 for PTEN. In tumors with low pre-decitabine tumor suppressor gene scores (≤150), expression was higher post-treatment in 8 of 8 cases for FHIT (*P* = 0.014), 7 of 17 for WWOX (*P* = 0.0547), 7 of 12 for FUS1 (*P* = 0.0726), and 1 of 16 for PTEN (*P* = 0.2034). If FHIT, WWOX and FUS1 were considered together, median pre- versus post-decitabine scores were 60 versus 100 (*P* = 0.0002). Overall, tumor suppressor gene expression change did not correlate with LINE-1 demethylation, although tumors converting from negative to positive had a median decrease in LINE-1 methylation of 24%, compared to 6% in those not converting (*P* = 0.069). Five of 15 fully evaluable patients had reductions in tumor diameter (range 0.2% to 33.4%). Of these, three had simultaneous increases in three tumor suppressor genes (including the two patients with the greatest tumor regression) compared to 2 of 10 with tumor growth (*P* = 0.25).

**Conclusions:**

In tumors with low tumor suppressor gene expression, decitabine may be associated with increased expression of the tumor suppressor genes FHIT, FUS1, and WWOX, but not PTEN.

## Background

Several tumor suppressor genes have now been described, and tumor suppressor gene silencing by mutation, deletion, or hypermethylation [1,2] is an important component of tumorigenesis. Treatment of cell lines or xenograft-bearing animals with the demethylating agents decitabine and 5-azacytidine has been reported to increase expression of a variety of tumor suppressor genes [3-6]. Decitabine may upregulate gene expression through both methylation-dependent and methylation-independent mechanisms [1,2].

Decitabine is active clinically in some hematologic malignancies [7]. Administration of low doses of decitabine daily for multiple successive days may be most effective against hematological malignancies, and is also particularly likely to induce DNA demethylation [7].

In patients with refractory malignancies receiving low dose decitabine on days 1 to 5 ± days 8 to 12 each cycle, we biopsied tumors before day 1 and on day 12 of cycle 1, and found in patient tumors that decitabine decreased methylation of the long interspersed nuclear element-1 (LINE-1) repetitive DNA element (as a surrogate for global DNA methylation) [8], while it increased tumor expression of the copper transport protein-1 (CTR1, a copper/platinum transporter) [8], the Ras homolog gene family member A (RhoA, an endocytosis regulator) [9], and the reduced folate carrier-1 (RFC1, a folate transporter) [9]. In pre-decitabine tumor samples, expression of CTR1 [8] and RhoA [9] was lower and LINE-1 methylation tended to be higher in patients who were ≤3 months versus >3 months beyond most recent prior therapy [8], and LINE-1 methylation correlated inversely with expression of CTR1 [8] and RhoA [9].

Based on our observations with CTR1 [8], LINE-1 [8], and RhoA [9], we then investigated whether expression of selected tumor suppressor genes would vary with time from last treatment, with LINE-1 methylation and with decitabine treatment. The tumor suppressor genes assessed were fragile histidine triad (*FHIT*), WW domain-containing oxidoreductase (*WWOX*), fused in sarcoma-1 (*FUS1*) and phosphatase and tensin homolog (*PTEN*).

*FHIT* is a proapoptotic tumor suppressor gene that encodes the fragile histidine triad protein (FHIT, also known as bis-(5’-adenosyl) triphosphatase), and *FHIT* inactivation or loss occurs in many tumor types [10]. Loss of FHIT leads to alterations in the DNA damage response checkpoint, resulting in increased DNA instability [11]. Loss of *FHIT* expression is commonly associated with hypermethylation of the gene, and frequent *FHIT* hypermethylation has been reported in hepatocellular carcinomas [12], and in carcinomas of the larynx [13], breast [14], lung [14], cervix [15], vulva [16], and kidney [17]. The demethylating agent decitabine may increase expression of FHIT in cancer cell lines [3].

*WWOX* is a large gene spanning the chromosomal fragile site 16D [18]. It encodes the protein WW domain-containing oxidoreductase (WWOX) which may play a role in apoptosis [19], cell metabolism [20], and modulation of the activity of multiple interacting transcription factors [21]. WWOX is generally strongly expressed in various normal tissues [22], but its expression by immunohistochemistry (IHC) is absent or weak in many cancers arising from tissues that generally express WWOX, including cancers of the breast [18], ovary [23], bladder [24], and esophagus [25], and in leukemias [19]. Exposure to carcinogens such as cigarette smoking extract can lead to downregulation of *WWOX* expression [24]. *WWOX* downregulation has been noted to occur via promoter methylation in various malignancies [25-27], and decitabine may restore WWOX expression [3,4].

The *FUS1* gene (also known as tumor suppressor candidate 2 or TUSC2) is located in the chromosomal 3p21.3 region. In lung cancers and various other malignancies, this chromosomal region is frequently deleted and *FUS1/TUSC2* expression is often lost [28]. *FUS1* functions as a tumor suppressor gene by inducing apoptosis through activation of the intrinsic mitochondrial-dependent and Apaf-1-associated pathways [28]. *FUS1/TUSC2* may be hypermethylated in cancers of the head and neck, and decitabine may reverse this hypermethylation [29]. In breast cancer cell lines, decitabine increased expression of *FUS1/TUSC2* despite lack of gene methylation [30], in keeping with the known ability of decitabine to increase gene expression through both methylation-dependent and methylation-independent mechanisms [1,2].

*PTEN* functions as a tumor suppressor gene by negatively regulating the Akt pathway, and it is one of the most frequently inactivated tumor suppressor genes in human cancers [31]. PTEN hypermethylation has been reported to be common in several tumor types [32-35]. Demethylating agents have been reported to restore PTEN expression in cell lines with hypermethylated PTEN [5,6]. However, PTEN protein expression did not change with decitabine exposure in ovarian cancer cell lines [36], and the role of promoter hypermethylation in silencing PTEN expression is not clear-cut. While the above studies suggested a role for PTEN promoter hypermethylation, several other studies across a range of malignancies have failed to detect significant PTEN promoter methylation [37-40]. It has been noted that the PTENP1 pseudogene (that has 98% homology with PTEN) is frequently hypermethylated in tumors and cell lines, while PTEN is not, and reports of methylation of PTEN have been attributed by some authors to misinterpretations of hypermethylation of PTENP1 [38].

## Results

### Patient characteristics

Patient characteristics are presented in more detail in an earlier publication on this patient group [8]. Patient numbers varied slightly between tumor suppressor genes since insufficient biopsy material was available for some assessments. Twenty-five of 31 patients who were initially entered on our decitabine phase I clinical trial [8] had sufficient tissue to permit at least one IHC assessment (either pre- or post-decitabine) of at least one of the four tumor suppressor genes of interest. These 25 patients included 14 males and 11 females, with a median (range) age of 53 (20 to 75) years. Tumor types included cancers of the breast (four patients), kidney (three), head and neck (three, including one adenocystic carcinoma), lung (one), stomach (one), and appendix (one), malignant melanomas (four), thymic neoplasms (three), neuroendocrine tumors (two), lymphomas (two), and desmoplastic tumor (one). Patients had received a median (range) of five (1 to 14) prior systemic regimens and a median (range) of two (0 to 6) prior targeted agents.

### Tumor suppressor gene immunohistochemistry scores versus time from last therapy

Comparing patients undergoing pre-decitabine tumor biopsy ≤3 months after last prior chemotherapy or targeted therapy to those undergoing initial biopsy >3 months after last therapy are presented, PTEN scores were significantly higher in patients with longer time intervals since last treatment (*P* = 0.007), and there was a trend towards higher FUS1 scores in later biopsies (*P* = 0.15), while there was no association of FHIT and WWOX scores with time from last treatment.

### Tumor suppressor gene immunohistochemistry scores versus LINE-1 methylation

Tumor suppressor gene scores did not correlate significantly with LINE-1 methylation in pre-decitabine tumor samples, nor in pre-and post-decitabine samples combined (Table 1). However, if pre-decitabine scores for FHIT, FUS1 and WWOX were considered together, tumors with IHC scores = 0 for one of these genes (eight observations) had significantly higher LINE-1 methylation than did tumors with IHC scores >0 (58 observations) (median 61.6% versus 45.4%, *P* = 0.0481).

**Table 1:** Correlation of tumor suppressor gene encoded proteins with LINE-1 methylation (pre- and post-decitabine)

**Protein**	**n**	**Spearman r**	***P***
FHIT	41	−0.23	0.15
WWOX	44	0.09	0.57
FUS1	41	0.15	0.35
PTEN	40	0.03	0.83

FHIT, fragile histidine triad; FUS1, fused in sarcoma-1; LINE-1, long interspersed nuclear element-1; PTEN, phosphatase and tensin homolog; WWOX, WW domain-containing oxidoreductase.

### Decitabine effect on tumor suppressor gene immunohistochemistry scores

**Figure 1:**
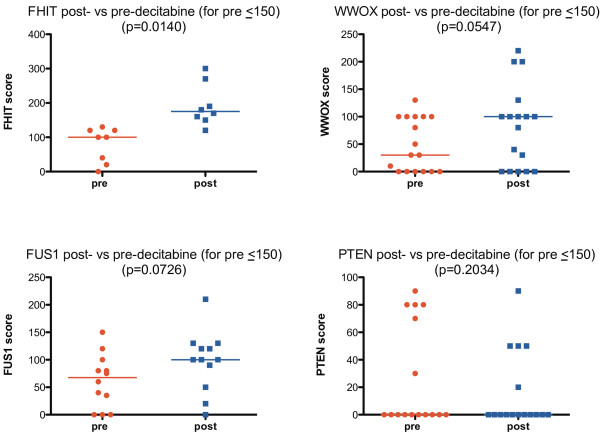
**Change in immunohistochemistry scores for fragile histidine triad (FHIT), WW domain-containing oxidoreductase (WWOX), fused in sarcoma-1 (FUS1) and phosphatase and tensin homolog (PTEN) with decitabine for tumors with scores that were initially low (≤150).** By Wilcoxon matched pairs signed rank tests, there was a significant increase in FHIT scores with decitabine (post- versus pre-decitabine median scores 175 versus 100, *P* = 0.0140), with a strong trend towards an increase in WWOX (post- versus pre-decitabine median scores 100 versus 30, *P* = 0.0547) and FUS1 (post- versus pre-decitabine median scores 100 versus 67.5, *P* = 0.0726), but with no major change in PTEN (post- versus pre-decitabine scores 0 versus 0, *P* = 0.2034). Medians are indicated by horizontal lines on the graphs.

For tumors with initially low tumor suppressor gene IHC expression (scores ≤150), expression was higher post-decitabine than pre-decitabine for FHIT (*P* = 0.0140), with a trend to higher expression post-decitabine for WWOX (*P* = 0.0547) and FUS1 (*P* = 0.0726), and little effect for PTEN (*P* = 0.2034) (Table 2, Figure 1). Overall, for pre-decitabine scores of ≤150, there was an increase in the score post-decitabine in 8 of 8 cases for FHIT, 7 of 17 for WWOX, 7 of 12 for FUS1, but only 1 of 16 for PTEN. The proportion of cases with a post-decitabine increase was significantly higher for FHIT, WWOX and FUS1 combined than for PTEN (*P* = 0.003 by Fisher’s exact test). If FHIT, WWOX and FUS1 were considered together then, for cases with initial scores ≤150, the median scores pre- and post-decitabine were 60 and 100, respectively (*P* = 0.0002).

**Table 2:** Median tumor suppressor gene encoded protein scores post- versus pre-decitabine for patients with pre-decitabine scores (≤150), and number of patients with an increase, no change or decrease in scores with decitabine

**Protein**	**n**	**Score pre-decitabine**	**Score post-decitabine**	***P***	**Number with increase in score**	**Number with no change in score**	**Number with decrease in score**
FHIT	8	100	175	0.014	8	0	0
WWOX	17	30	100	0.05	7	8	2
FUS1	12	67.5	100	0.07	7	2	3
PTEN	16	0	0	0.20	1	10	5
FHIT, WWOX or FUS1	37	60	100	0.0002	22	10	5

FHIT, fragile histidine triad; FUS1, fused in sarcoma-1; PTEN, phosphatase and tensin homolog; WWOX, WW domain-containing oxidoreductase.

Of those tumors that initially stained negatively for a tumor suppressor gene (pre-decitabine score = 0), there was conversion to positive staining post-decitabine for FHIT in 1 of 1 patients, for WWOX in 3 of 6 patients, for FUS1 in 2 of 3 patients, and for PTEN in 1 of 10 patients.

### Post-decitabine changes in tumor suppressor gene immunohistochemistry scores versus percentage changes in LINE-1 DNA methylation

While there was a weak negative correlation between percentage change in LINE-1 methylation and IHC score for each tumor suppressor gene, this did not achieve significance (Table 3). If we only considered tumors that initially stained negatively for a gene, there was a stronger trend (*P* = 0.069) for ones that converted from negative to positive staining with decitabine to have a greater decrease in LINE-1 methylation with decitabine (median decrease of 24% in LINE-1 methylation) than for those that did not convert (median decrease in LINE-1 methylation of 6%).

**Table 3:** Change in tumor suppressor gene encoded protein score versus percentage change in LINE-1 methylation with decitabine

**Protein**	**n**	**Spearman r**	***P***
FHIT	16	−0.34	0.19
WWOX	19	−0.04	0.87
FUS1	16	−0.03	0.91
PTEN	15	−0.26	0.36
Any tumor suppressor gene	67	−0.11	0.39

FHIT, fragile histidine triad; FUS1, fused in sarcoma-1; PTEN, phosphatase and tensin homolog; WWOX, WW domain-containing oxidoreductase.

### Tumor suppressor gene immunohistochemistry score changes and tumor regression

Of the 25 patients included, both change in tumor size with the first cycle of therapy and change in IHC scores for all four tumor suppressor genes could be assessed in 15 patients. Of these, 10 patients had immediate tumor growth, while five patients had a reduction in tumor diameter of 0.2%, 2%, 4.7%, 22.1% and 33.4%, respectively. Among the five with any degree of tumor regression, all five had increases in FHIT scores, three had increases in WWOX scores, three had increases in FUS1 scores, and one had an increase in PTEN score. All five had an increase in at least one tumor suppressor gene, and three had simultaneous increases in three tumor suppressor genes (including the two patients with the greatest tumor regression), compared to 2 of 10 fully evaluable patients with tumor growth who had an increase in expression of three tumor suppressor genes (*P* = 0.25 by Fisher’s exact test). Median (range) number of tumor suppressor genes increasing with decitabine was three (1 to 3) in those with tumor regression versus two (0 to 3) in fully evaluable patients with tumor growth (*P* = 0.34).

## Discussion

In our phase I trial of the demethylating agent decitabine we had previously reported that percentage LINE-1 DNA methylation correlated inversely with expression of the copper/platinum transporter CTR1 and the endocytosis regulator RhoA, and that decitabine treatment was associated with an increase in expression of CTR1, RhoA and of the folate carrier RFC1 in patients in whom expression was initially low [8,9]. Early phase clinical trials also suggest that addition of decitabine to a platinum agent may partially reverse platinum-resistance in advanced ovarian carcinomas [41-43]. Hence, there is interest in further exploring the role of decitabine and other demethylating agents as resistance-modulating therapies.

In this further assessment of patients included in our phase I trial of low-dose single-agent decitabine, we assessed the impact of decitabine on expression of selected tumor suppressor genes. Loss of tumor suppressor gene function through gene deletion, mutation, or silencing (through promoter hypermethylation or other means) is important in tumorigenesis, but replacing lost tumor suppressor gene function is difficult. Attempts at tumor suppressor gene replacement by adenovirus-mediated gene therapy have met with modest early indications of success [44-46], although administration has generally been by local injection or similar means, and systemic delivery to widespread metastases is challenging. However, it has recently been demonstrated that intravenous administration of the tumor suppressor gene FUS1/TUSC2 in DOTAP nanoparticles is capable of systemic delivery to disseminated disease [47], and this approach is undergoing further investigation.

Our results with decitabine suggest that demethylating agents may be capable of upregulating expression of selected tumor suppressor genes in some patients. Low patient numbers have limited statistical power but, despite this, there was a statistically significant increase in expression of FHIT following decitabine, with a strong statistical trend towards increase in expression of WWOX and FUS1, and combining data for FHIT, WWOX and FUS1 indicated a significant increase in IHC expression of these three tumor suppressor genes together. This is sufficient to encourage further assessment of the ability of demethylating agents to restore tumor suppressor gene function in situations where it has been decreased or lost due to promoter hypermethylation. For each of these tumor suppressor genes, available published data suggest that promoter methylation may be an important cause of loss of gene function [3,4,12-17,25-27,29,48,49], although we did not have sufficient residual tissue to permit us to assess this in our study, and it is probable that other mechanisms such as gene deletion or mutation were responsible for low expression in a proportion of the tumors. We also did not have sufficient tissue available to assess whether increase in tumor suppressor gene protein expression with decitabine was associated with reduction in tumor suppressor gene DNA methylation.

There was no apparent increase in PTEN IHC scores with decitabine treatment, and changes in PTEN scores were significantly lower than changes in scores for the other tumor suppressor genes. This is in keeping with the indication from many publications that promoter methylation may not be an important cause of loss of PTEN expression [37-39], and in keeping with the hypothesis that an apparent role for PTEN promoter methylation may be due to misinterpretation of data arising from methylation of the closely related pseudogene PTENP1 [38]. An additional potential explanation for the lack of impact of decitabine on PTEN is that, for at least some genes, gene reactivation in response to decitabine requires chromatin remodeling in addition to DNA demethylation if histone modification is playing a role in gene silencing [50].

All five patients experiencing tumor regression with decitabine had an increase in the IHC score of at least one tumor suppressor gene, and three (including the two with the greatest regression) had an increase in IHC scores of three tumor suppressor genes, compared to 2 of 10 fully evaluable patients who had tumor growth who had an increase in IHC scores for three tumor suppressor genes, and the median number of tumor suppressor genes that increased with decitabine was three in patients with tumor regression versus two in patients with tumor growth. Further assessment will be needed to determine if change in tumor suppressor genes with decitabine impacts the probability of achieving tumor regression.

## Conclusions

Overall, our data add further evidence that exploration of demethylating agents in solid tumors may be of interest. It may be of particular interest to explore them in tumors that are demonstrated to have low expression of tumor suppressor genes in association with promoter methylation.

## Methods

This study was approved by the MD Anderson Cancer Center Research Ethics Board, and recruited consenting patients with advanced malignancies, tumors that could be safely biopsied, and organ function meeting eligibility requirements [8]. Decitabine was supplied under a Collaborative and Research Development Agreement by the National Cancer Institute Division of Cancer Treatment and Diagnosis. Decitabine doses of 2.5, 5, or 10 mg/m^2^/day on days 1 to 5 and 8 to 12 each 4-week cycle or 15 or 20 mg/m^2^/day on days 1 to 5 each cycle were administered over 1 hour, with filgrastim added at higher doses [8].

Tumor biopsies were performed pre-decitabine and on day 12, cycle 1 [8]. Formalin-fixed, paraffin-embedded tissue sections (5 μm thick) were deparaffinized in Xylene (10 minutes × 3), followed by hydration in sequenced graded alcohols (5 minutes each). Heat-induced epitope retrieval was performed in DAKO solution for 30 minutes at 121°C, followed by 10 minutes at 90°C using a Decloaking chamber (Biocare, Concord, CA), followed by a 30-minute cool-down. Prior to antibody immunostaining, endogenous peroxidase activity was blocked with 3% hydrogen peroxide in methanol for 30 minutes. To block non-specific antibody binding, tissues were incubated in 10% fetal bovine serum/Tris-buffered saline with Tween 20 for 30 minutes. Primary incubation antibodies are presented in Table 4. This was followed by incubation with Envision plus labeled polymer, anti-rabbit-horseradish peroxidase antibody (DAKO, Carpinteria, CA) for 30 minutes at room temperature. The FUS1 protocol was as previously described [47]. Time monitoring staining development was performed with diaminobenzidine, using a reliable positive control sample. Slides were counterstained with hematoxylin, dehydrated, cleared and mounted.

**Table 4:** Antibodies used for immunohistochemistry

**Protein**	**Source**	**Dilution**
FHIT	Thermo Scientific, Waltham, MA, USA	1:100
WWOX	Abcam, Cambridge, MA, USA	1:100
FUS1	Homemade, rabbit polyclonal	1:400
PTEN	Cell Signaling, Danvers, MA, USA	1:75

FHIT, fragile histidine triad; FUS1, fused in sarcoma-1; PTEN, phosphatase and tensin homolog; WWOX, WW domain-containing oxidoreductase.

Participating pathologist MIN scored staining intensity as 0 to 3+ and generated IHC scores of 0 to 300 by multiplying the percent of tumor cells staining by the staining intensity. For PTEN, we assessed both cytoplasmic and nuclear staining scores, but report only on the cytoplasmic scores since results were similar for the two staining sites. Change in IHC score was defined as the day 12 score minus the day 1 score.

As previously reported, the LINE-1 assay was used to define percentage of DNA CpG islands that were methylated, as a surrogate for global DNA methylation [8,51]. Change in LINE-1 methylation was defined as the day 12 value minus the day 1 value divided by the day 1 value.

While low patient numbers limited statistical power, GraphPad Prism 5.0 (GraphPad Software, San Diego, CA) was used to assess non-parametric two-tailed statistics (Spearman tests for correlations, Wilcoxon signed rank tests for paired comparisons, and Mann–Whitney tests and Fisher exact tests for comparisons of two groups).

## Abbreviations

CTR1: copper transport protein-1; FHIT: fragile histidine triad; FUS1: fused in sarcoma-1; IHC: immunohistochemistry; LINE-1: long interspersed nuclear element-1; PTEN: phosphatase and tensin homolog; PTENP1: phosphatase and tensin homolog pseudogene; RFC1: reduced folate carrier-1; RhoA: Ras homolog gene family member A; TUSC2: tumor suppressor candidate 2; WWOX: WW domain-containing oxidoreductase.

## Competing interests

The authors declare that they have no competing interests.

## Authors’ contributions

DJS designed and oversaw the overall study, analyzed the data and drafted the manuscript. MIN performed all immunohistochemistry on tumor samples. JJ assessed LINE-1 methylation of tumor samples. DH contributed to patient recruitment. SG oversaw tumor biopsies. MA contributed to WWOX studies. JPI oversaw LINE-1 methylation studies. RK oversaw patient recruitment. IIW oversaw tumor sample collection, storage, retrieval and immunohistochemistry. All authors read and approved the final manuscript.

## References

[B1] Oki Y Aoki E Issa JP Decitabine - bedside to bench Crit Rev Oncol Hematol 2007 61 140 152 17023173 10.1016/j.critrevonc.2006.07.010

[B2] Ghoshal K Bai S DNA methyltransferases as targets for cancer therapy Drugs Today (Barc) 2007 43 395 422 17612710 10.1358/dot.2007.43.6.1062666

[B3] Cantor JP Iliopoulos D Rao AS Druck T Semba S Han SY McCorkell KA Lakshman TV Collins JE Wachsberger P Friedberg JS Huebner K Epigenetic modulation of endogenous tumor suppressor expression in lung cancer xenografts suppresses tumorigenicity Int J Cancer 2007 120 24 31 17019711 10.1002/ijc.22073

[B4] Iliopoulos D Fabbri M Druck T Qin HR Han SY Huebner K Inhibition of breast cancer cell growth in vitro and in vivo: effect of restoration of Wwox expression Clin Cancer Res 2007 13 268 274 17200365 10.1158/1078-0432.CCR-06-2038

[B5] Phuong NT Kim SK Lim SC Kim HS Kim TH Lee KY Ahn SG Yoon JH Kang KW Role of PTEN promoter methylation in tamoxifen-resistant breast cancer cells Breast Cancer Res Treat 2011 130 73 83 21170675 10.1007/s10549-010-1304-2

[B6] Gravina GL Biordi L Martella F Flati V Ricevuto E Ficorella C Tombolini V Festuccia C Epigenetic modulation of PTEN expression during antiandrogenic therapies in human prostate cancer Int J Oncol 2009 35 1133 1139 19787268 10.3892/ijo_00000429

[B7] Issa JP Gharibyan V Cortes J Jelinek J Morris G Verstovsek S Talpaz M Garcia-Manero G Kantarjian HM Phase II study of low-dose decitabine in patients with chronic myelogenous leukemia resistant to imatinib mesylate J Clin Oncol 2005 23 3948 3956 15883410 10.1200/JCO.2005.11.981

[B8] Stewart DJ Issa JP Kurzrock R Nunez MI Jelinek J Hong D Oki Y Guo Z Gupta S Wistuba II Decitabine effect on tumor global DNA methylation and other parameters in a phase I trial in refractory solid tumors and lymphomas Clin Cancer Res 2009 15 3881 3888 19470736 10.1158/1078-0432.CCR-08-2196

[B9] Stewart DJ Nunez MI Jelinek J Hong D Gupta S Issa JP Wistuba II Kurzrock R Decitabine impact on the endocytosis regulator RhoA, the folate carriers RFC1 and FOLR1, and the glucose transporter GLUT4 in human tumors Clin Epigenetics 2014 6 1 2 24401732 10.1186/1868-7083-6-2 PMC3895853

[B10] Ishii H Ozawa K Furukawa Y Alteration of the fragile histidine triad gene early in carcinogenesis: an update J Exp Ther Oncol 2003 3 291 296 14678517 10.1111/j.1533-869x.2003.01101.x

[B11] Pichiorri F Palumbo T Suh SS Okamura H Trapasso F Ishii H Huebner K Croce CM Fhit tumor suppressor: guardian of the preneoplastic genome Future Oncol 2008 4 815 824 19086848 10.2217/14796694.4.6.815 PMC3400506

[B12] Zhang X Li HM Liu Z Zhou G Zhang Q Zhang T Zhang J Zhang C Loss of heterozygosity and methylation of multiple tumor suppressor genes on chromosome 3 in hepatocellular carcinoma J Gastroenterol 2013 48 132 143 22766745 10.1007/s00535-012-0621-0

[B13] Paluszczak J Misiak P Wierzbicka M Wozniak A Baer-Dubowska W Frequent hypermethylation of DAPK, RARbeta, MGMT, RASSF1A and FHIT in laryngeal squamous cell carcinomas and adjacent normal mucosa Oral Oncol 2011 47 104 107 21147548 10.1016/j.oraloncology.2010.11.006

[B14] Zochbauer-Muller S Fong KM Maitra A Lam S Geradts J Ashfaq R Virmani AK Milchgrub S Gazdar AF Minna JD 5’ CpG island methylation of the FHIT gene is correlated with loss of gene expression in lung and breast cancer Cancer Res 2001 61 3581 3585 11325823

[B15] Ki KD Lee SK Tong SY Lee JM Song DH Chi SG Role of 5’-CpG island hypermethylation of the FHIT gene in cervical carcinoma J Gynecol Oncol 2008 19 117 122 19471558 10.3802/jgo.2008.19.2.117 PMC2676455

[B16] Stephen JK Chen KM Raitanen M Grenman S Worsham MJ DNA hypermethylation profiles in squamous cell carcinoma of the vulva Int J Gynecol Pathol 2009 28 63 75 19047905 10.1097/PGP.0b013e31817d9c61 PMC2605778

[B17] Kvasha S Gordiyuk V Kondratov A Ugryn D Zgonnyk YM Rynditch AV Vozianov AF Hypermethylation of the 5’CpG island of the FHIT gene in clear cell renal carcinomas Cancer Lett 2008 265 250 257 18378390 10.1016/j.canlet.2008.02.036

[B18] Nunez MI Ludes-Meyers J Abba MC Kil H Abbey NW Page RE Sahin A Klein-Szanto AJ Aldaz CM Frequent loss of WWOX expression in breast cancer: correlation with estrogen receptor status Breast Cancer Res Treat 2005 89 99 105 15692750 10.1007/s10549-004-1474-x PMC4145848

[B19] Cui Z Lin D Cheng F Luo L Kong L Xu J Hu J Lan F The role of the WWOX gene in leukemia and its mechanisms of action Oncol Rep 2013 29 2154 2162 23525648 10.3892/or.2013.2361

[B20] Dayan S O’Keefe LV Choo A Richards RI Common chromosomal fragile site FRA16D tumor suppressor WWOX gene expression and metabolic reprograming in cells Genes Chromosomes Cancer 2013 52 823 831 23765596 10.1002/gcc.22078

[B21] Aqeilan RI Croce CM WWOX in biological control and tumorigenesis J Cell Physiol 2007 212 307 310 17458891 10.1002/jcp.21099

[B22] Nunez MI Ludes-Meyers J Aldaz CM WWOX protein expression in normal human tissues J Mol Histol 2006 37 115 125 16941225 10.1007/s10735-006-9046-5 PMC4144810

[B23] Nunez MI Rosen DG Ludes-Meyers JH Abba MC Kil H Page R Klein-Szanto AJ Godwin AK Liu J Mills GB Aldaz CM WWOX protein expression varies among ovarian carcinoma histotypes and correlates with less favorable outcome BMC Cancer 2005 5 64 15982416 10.1186/1471-2407-5-64 PMC1173095

[B24] Yang W Cui S Ma J Lu Q Kong C Liu T Sun Z Cigarette smoking extract causes hypermethylation and inactivation of WWOX gene in T-24 human bladder cancer cells Neoplasma 2012 59 216 223 22248280 10.4149/neo_2012_028

[B25] Guo W Wang G Dong Y Guo Y Kuang G Dong Z Decreased expression of WWOX in the development of esophageal squamous cell carcinoma Mol Carcinog 2013 52 265 274 22213016 10.1002/mc.21853

[B26] Pluciennik E Nowakowska M Wujcicka WI Sitkiewicz A Kazanowska B Zielińska E Bednarek AK Genetic alterations of WWOX in Wilms’ tumor are involved in its carcinogenesis Oncol Rep 2012 28 1417 1422 22842668 10.3892/or.2012.1940

[B27] Baykara O Demirkaya A Kaynak K Tanju S Toker A Buyru N WWOX gene may contribute to progression of non-small-cell lung cancer (NSCLC) Tumour Biol 2010 31 315 320 20480411 10.1007/s13277-010-0039-3

[B28] Ji L Roth JA Tumor suppressor FUS1 signaling pathway J Thorac Oncol 2008 3 327 330 18379348 10.1097/JTO.0b013e31816bce65 PMC3370667

[B29] Demokan S Chuang AY Chang X Khan T Smith IM Pattani KM Dasgupta S Begum S Khan Z Liegeois NJ Westra WH Sidransky D Koch W Califano JA Identification of guanine nucleotide-binding protein gamma-7 as an epigenetically silenced gene in head and neck cancer by gene expression profiling Int J Oncol 2013 42 1427 1436 23403885 10.3892/ijo.2013.1808 PMC3981008

[B30] da Costa PE Cavalli LR Rainho CA Evidence of epigenetic regulation of the tumor suppressor gene cluster flanking RASSF1 in breast cancer cell lines Epigenetics 2011 6 1413 1424 22139571 10.4161/epi.6.12.18271 PMC3256331

[B31] Hollander MC Blumenthal GM Dennis PA PTEN loss in the continuum of common cancers, rare syndromes and mouse models Nat Rev Cancer 2011 11 289 301 21430697 10.1038/nrc3037 PMC6946181

[B32] Yin L Cai WJ Liu CX Chen YZ Hu JM Jiang JF Li HA Cui XB Chang XY Zhang WJ Sun K Li F Analysis of PTEN methylation patterns in soft tissue sarcomas by MassARRAY spectrometry PLoS One 2013 8 e62971 23690972 10.1371/journal.pone.0062971 PMC3656904

[B33] Kuo LT Kuo KT Lee MJ Wei CC Scaravilli F Tsai JC Tseng HM Kuo MF Tu YK Correlation among pathology, genetic and epigenetic profiles, and clinical outcome in oligodendroglial tumors Int J Cancer 2009 124 2872 2879 19330828 10.1002/ijc.24303

[B34] Shetty PJ Pasupuleti N Chava S Nasaruddin K Hasan Q Altered transcription and expression of PTEN in breast tumors: is it regulated by hypermethylation? Breast Dis 2011 33 27 33 21846942 10.3233/BD-2010-0312

[B35] Yang HJ Liu VW Wang Y Tsang PC Ngan HY Differential DNA methylation profiles in gynecological cancers and correlation with clinico-pathological data BMC Cancer 2006 6 212 16928264 10.1186/1471-2407-6-212 PMC1560388

[B36] Schondorf T Ebert MP Hoffmann J Becker M Moser N Pur S Göhring UJ Weisshaar MP Hypermethylation of the PTEN gene in ovarian cancer cell lines Cancer Lett 2004 207 215 220 15072831 10.1016/j.canlet.2003.10.028

[B37] Kawaguchi K Oda Y Saito T Takahira T Yamamoto H Tamiya S Iwamoto Y Tsuneyoshi M Genetic and epigenetic alterations of the PTEN gene in soft tissue sarcomas Hum Pathol 2005 36 357 363 15891996 10.1016/j.humpath.2005.01.017

[B38] Hesson LB Packham D Pontzer E Funchain P Eng C Ward RL A reinvestigation of somatic hypermethylation at the PTEN CpG island in cancer cell lines Biological Procedures Online 2012 14 5 22490388 10.1186/1480-9222-14-5 PMC3342897

[B39] Yu J Ni M Xu J Zhang H Gao B Gu J Chen J Zhang L Wu M Zhen S Zhu J Methylation profiling of twenty promoter-CpG islands of genes which may contribute to hepatocellular carcinogenesis BMC Cancer 2002 2 29 12433278 10.1186/1471-2407-2-29 PMC139988

[B40] Tamura G Promoter methylation status of tumor suppressor and tumor-related genes in neoplastic and non-neoplastic gastric epithelia Histol Histopathol 2004 19 221 228 14702190 10.14670/HH-19.221

[B41] Matei D Fang F Shen C Schilder J Arnold A Zeng Y Berry WA Huang T Nephew KP Epigenetic resensitization to platinum in ovarian cancer Cancer Res 2012 72 2197 2205 22549947 10.1158/0008-5472.CAN-11-3909 PMC3700422

[B42] Fu S Hu W Iyer R Kavanagh JJ Coleman RL Levenback CF Sood AK Wolf JK Gershenson DM Markman M Hennessy BT Kurzrock R Bast RC Jr Phase 1b-2a study to reverse platinum resistance through use of a hypomethylating agent, azacitidine, in patients with platinum-resistant or platinum-refractory epithelial ovarian cancer Cancer 2011 117 1661 1669 21472713 10.1002/cncr.25701 PMC3062960

[B43] Fang F Balch C Schilder J Breen T Zhang S Shen C Li L Kulesavage C Snyder AJ Nephew KP Matei DE A phase 1 and pharmacodynamic study of decitabine in combination with carboplatin in patients with recurrent, platinum-resistant, epithelial ovarian cancer Cancer 2010 116 4043 4053 20564122 10.1002/cncr.25204 PMC2930033

[B44] Guan YS Liu Y He Q Li X Yang L Hu Y La Z p53 gene therapy in combination with transcatheter arterial chemoembolization for HCC: one-year follow-up World J Gastroenterol 2011 17 2143 2149 21547136 10.3748/wjg.v17.i16.2143 PMC3084402

[B45] Swisher SG Roth JA Nemunaitis J Lawrence DD Kemp BL Carrasco CH Connors DG El-Naggar AK Fossella F Glisson BS Hong WK Khuri FR Kurie JM Lee JJ Lee JS Mack M Merritt JA Nguyen DM Nesbitt JC Perez-Soler R Pisters KM Putnam JB Jr Richli WR Savin M Schrump DS Shin DM Shulkin A Walsh GL Wait J Weill D Waugh MK Adenovirus-mediated p53 gene transfer in advanced non-small-cell lung cancer J Natl Cancer Inst 1999 91 763 771 10328106 10.1093/jnci/91.9.763

[B46] Clayman GL El-Naggar AK Lippman SM Henderson YC Frederick M Merritt JA Zumstein LA Timmons TM Liu TJ Ginsberg L Roth JA Hong WK Bruso P Goepfert H Adenovirus-mediated p53 gene transfer in patients with advanced recurrent head and neck squamous cell carcinoma J Clin Oncol 1998 16 2221 2232 9626224 10.1200/JCO.1998.16.6.2221

[B47] Lu C Stewart DJ Lee JJ Ji L Ramesh R Jayachandran G Nunez MI Wistuba II Erasmus JJ Hicks ME Grimm EA Reuben JM Baladandayuthapani V Templeton NS McMannis JD Roth JA Phase I clinical trial of systemically administered TUSC2(FUS1)-nanoparticles mediating functional gene transfer in humans PLoS One 2012 7 e34833 22558101 10.1371/journal.pone.0034833 PMC3338819

[B48] Li W Deng J Jiang P Tang J Association of 5’-CpG island hypermethylation of the FHIT gene with lung cancer in southern-central Chinese population Cancer Biol Ther 2010 10 997 1000 20814237 10.4161/cbt.10.10.13231

[B49] Kim JS Kim JW Han J Shim YM Park J Kim DH Cohypermethylation of p16 and FHIT promoters as a prognostic factor of recurrence in surgically resected stage I non-small cell lung cancer Cancer Res 2006 66 4049 4054 16618724 10.1158/0008-5472.CAN-05-3813

[B50] Si J Boumber YA Shu J Qin T Ahmed S He R Jelinek J Issa JP Chromatin remodeling is required for gene reactivation after decitabine-mediated DNA hypomethylation Cancer Res 2010 70 6968 6977 20713525 10.1158/0008-5472.CAN-09-4474 PMC2932851

[B51] Yang AS Estecio MR Doshi K Kondo Y Tajara EH Issa JP A simple method for estimating global DNA methylation using bisulfite PCR of repetitive DNA elements Nucleic Acids Res 2004 32 e38 14973332 10.1093/nar/gnh032 PMC373427

